# Influence of Albumin Treatment Duration on Outcome of Terlipressin Therapy in Patients With HRS‐AKI

**DOI:** 10.1111/liv.70888

**Published:** 2026-09-30

**Authors:** Eva Maria Schleicher, Christian Labenz, Frank Erhard Uschner, Julian Pohl, Moritz Passenberg, Michael Praktiknjo, Marcus Maximilian Mücke, Henrik Karbannek, Nina Böhling, Karsten Große, Marlene Reincke, Sarah Schütte, Paul Jamme, Julian Cardinal von Widdern, Christoph Welsch, Jonel Trebicka, Cornelius Engelmann, Jassin Rashidi‐Alavijeh, Cristina Ripoll, Dominik Bettinger, the German cirrhosis study group, Johannes Chang, Maximilian Joseph Brol, Tony Bruns, Marko Damm, Jan Hendrik Harten, Christian Jansen, Alexander Queck, Christian Lange, Lauin Mustafa, Benjamin Maasoumy, Andreas Stallmach, Anna Teresa Volk, Alexander Zipprich

**Affiliations:** ^1^ Department of Internal Medicine I University Medical Center of the Johannes Gutenberg‐University Mainz Germany; ^2^ Department of Internal Medicine B University Hospital Münster Münster Germany; ^3^ Department of Hepatology and Gastroenterology Charité‐Universitätsmedizin Berlin Berlin Germany; ^4^ Department of Gastroenterology, Hepatology and Transplant Medicine University of Duisburg‐Essen Essen Germany; ^5^ Medical Clinic 1 University Hospital, Goethe‐University Frankfurt Frankfurt am Main Germany; ^6^ Department of Internal Medicine IV Jena University Hospital Jena Germany; ^7^ Department of Internal Medicine I Bonn University Hospital Bonn Germany; ^8^ Department of Internal Medicine III University Hospital RWTH Aachen Aachen Germany; ^9^ Department of Medicine II Medical Center University of Freiburg Freiburg Germany; ^10^ Department of Gastroenterology, Hepatology, Infectious Diseases and Endocrinology Hannover Medical School Hannover Germany; ^11^ Department of Internal Medicine II LMU University Hospital Munich Munich Germany; ^12^ Department of Internal Medicine I University Hospital Halle, Martin‐Luther‐University Halle‐Wittenberg Halle Germany

**Keywords:** acute kidney injury, albumin, ascites, cirrhosis, hepatorenal syndrome, HRS‐AKI, KDIGO, portal hypertension, terlipressin, volume expansion therapy

## Abstract

**Background and Aims:**

Volume expansion with albumin is recommended to establish a diagnosis of hepatorenal syndrome–acute kidney injury (HRS‐AKI). The optimal duration of albumin administration is debated and requires balancing the risk of overtreatment with the need for timely diagnosis and treatment. Therefore, this study aimed to compare the impact of different albumin treatment durations on terlipressin response and the prognosis of patients with HRS‐AKI treated with terlipressin and albumin.

**Methods:**

This multicenter, retrospective secondary analysis included patients with suspected HRS‐AKI who were treated with terlipressin and albumin at 12 German centers. One hundred forty five patients were available for further analysis. The cohort was dichotomized into patients treated with albumin for < 24 h and for 24–60 h before initiation of terlipressin. Patients were followed for treatment response to terlipressin, need for haemodialysis (HD), survival, or liver transplantation (LT).

**Results:**

Median MELD score of the cohort was 25 [IQR 21; 31]. During follow‐up, 59 patients (40.7%) achieved a complete response to treatment with terlipressin, and 96 patients (66.2%) died or received an LT. Response rates to terlipressin therapy did not differ between albumin treatment groups (< 24 h vs. 24–60 h). In multivariable competing risk models, treatment duration of albumin was not associated with any response to terlipressin, considering death and LT as competing events. Of note, albumin treatment duration did not affect HD−/LT‐free survival.

**Conclusion:**

Extending albumin treatment beyond 24 h had no impact on treatment response to terlipressin and albumin or HD‐/LT‐free survival in patients with HRS‐AKI.

**Clinical Trial Number:**

NCT 06161766

## Lay Summary

Volume expansion is necessary to confirm the diagnosis of HRS‐AKI, but it's unclear how long this initial treatment should last before moving on to other drugs like terlipressin. This study provides insights into the impact of treatment duration (< 24 h vs. 24–60 h) with albumin on treatment response and haemodialysis and transplantation‐free survival. No differences with regard to treatment response to terlipressin and albumin, haemodialysis and transplantation‐free survival were observed. These findings support individualized management strategies in volume expansion in patients with cirrhosis and acute kidney injury.

**Abbreviations**
AASLD: American Association for the Study of Liver Diseases
ACLF: acute‐on‐chronic liver failure
ADQI: acute disease quality initiative
AKI: acute kidney injury
ALD: alcohol‐related liver disease
BW: body weight
CI: confidence interval
CIF: cumulative incidence function
CRP: C‐reactive Protein
EASL: European Association for the Study of the Liver
EC: ethics committee
eGFR: estimated glomerular filtration rate
HCC: hepatocellular carcinoma
HD: Haemodialysis
HRS‐AKI: hepatorenal syndrome‐acute kidney injury
ICA: International club of ascites
INR: international normalized ratio
IQR: interquartile range
KDIGO: kidney disease improving global outcomes
LT: liver transplantation
MASLD: metabolic dysfunction‐associated steatotic liver disease
MELD: model of end‐stage liver disease
SCr: serum creatinine
TIPS: Transjugular intrahepatic portosystemic shunt
WBC: white blood cell count

## 1 Introduction

One of the most severe complications of cirrhosis is an acute deterioration of renal function known as hepatorenal syndrome‐acute kidney injury (HRS‐AKI). Diagnosing HRS‐AKI involves excluding other potential causes of acute kidney injury (AKI), such as hypovolemia, shock, nephrotoxic drugs, and intrinsic kidney disorders [1]. It refers to a functional renal impairment that occurs in patients with cirrhosis and ascites due to reduced renal perfusion secondary to hemodynamic alterations in the arterial circulation and activation of the endogenous vasoactive system [1, 2, 3]. Due to its high mortality, a rapid work‐up and diagnosis of its underlying cause are crucial to ensure timely diagnosis and treatment of HRS‐AKI. Recently, the Acute Disease Quality Initiative (ADQI) International Club of Ascites (ICA) consensus paper proposed changes to the diagnostic criteria, including a shorter time of albumin treatment to rule out hypovolemia from the previously recommended 48 h to a maximum of 24 h in order to avoid delays in the diagnosis of HRS‐AKI and impairments of prognosis [4]. Data on the optimal duration of albumin treatment prior to terlipressin initiation in HRS‐AKI remain limited. A recent randomized trial in patients with ACLF‐associated AKI suggested benefits of earlier terlipressin initiation after 12 h of albumin resuscitation [5]. However, recent evidence suggests that a significant proportion of patients respond to volume expansion within 24–48 h, challenging this new recommendation and sparking scientific debates [6, 7, 8]. Thus, determining the appropriate duration of albumin, particularly in those without response at 24 h, is a delicate balance. On one hand, one should avoid overtreatment with terlipressin, on the other hand, timely initiation of terlipressin, in case of HRS‐AKI, is required. Therefore, this study aimed to assess the influence of albumin treatment duration (< 24 h vs. 24–60 h) on (i) the treatment response to terlipressin, (ii) overall and liver transplant (LT)‐free survival, (iii) as well as the need for haemodialysis (HD).

## 2 Patients and Methods

### 2.1 Patient Cohort

This study represents a secondary analysis of a multicentre retrospective database that was originally established to evaluate the impact of pre‐existing chronic kidney disease (CKD) on outcomes in patients with HRS‐AKI [9]. For this multicentre, retrospective observational study, 490 patients treated with terlipressin and albumin for suspected HRS‐AKI, hospitalized between the 1st of January 2018, and the 31st of December 2022, from 12 tertiary centres in Germany were screened. Eligibility criteria were applied in two sequential steps. First, patients were selected according to the inclusion and exclusion criteria defined in the original study protocol and ClinicalTrials.gov registration (NCT06161766) [9]. Cirrhosis was diagnosed through histological examination or a combination of characteristic ultrasound findings, radiological imaging, endoscopic signs of portal hypertension, and medical history. Blood biochemical analysis was performed for all patients. To ensure a reliable assessment of kidney function, pre‐existing data on serum creatinine (SCr) and estimated glomerular filtration rate (eGFR) from at least 3 months before admission, in a stable condition, were assessed. Once a diagnosis of HRS‐AKI was established by the treating physician, patients received terlipressin and albumin, according to guidelines valid at the time of treatment [10, 11]. Patients were excluded if they fulfilled one or more of the following criteria: uncontrolled shock, cardiac cirrhosis (defined as the development of cirrhosis in the context of chronic heart failure due to a primary cardiac disease e.g., ischemic or hypertensive cardiomyopathy), the presence of hepatocellular carcinoma (HCC) outside of the Milan criteria, terlipressin treatment due to variceal bleeding, ongoing diuretic treatment, or missing data on outcome. Patients with TIPS were included only if ascites was detectable and therefore a disease‐modifying effect of TIPS on the course of cirrhosis could be excluded.

Second, for the present analysis focusing on the association between albumin treatment duration and response to terlipressin, additional analysis‐specific eligibility criteria were applied. As the original database lacked sufficient granularity regarding albumin administration before initiation and SCr at initiation of terlipressin therapy, participating centres were subsequently asked to provide additional data on these variables. Volume expansion was not standardized across centres and was performed according to local clinical practice and guideline recommendations in place at the time of treatment, given the study's retrospective, real‐world nature. Based on data availability and to ensure a valid volume exposure definition, a final selection step was applied, resulting in a reduced sample size compared with the overall cohort and the previously published analysis. Patients were excluded if one of the following criteria applied: (i) incomplete data regarding albumin treatment, (ii) albumin treatment for more than 60 h before terlipressin initiation, (iii) no documented longitudinal albumin administration prior to terlipressin therapy, including patients who received only a single baseline dose or had prior treatment at other hospitals. The final dataset consisted of 145 patients. The study flowchart is displayed in Figure 1.

**FIGURE 1:**
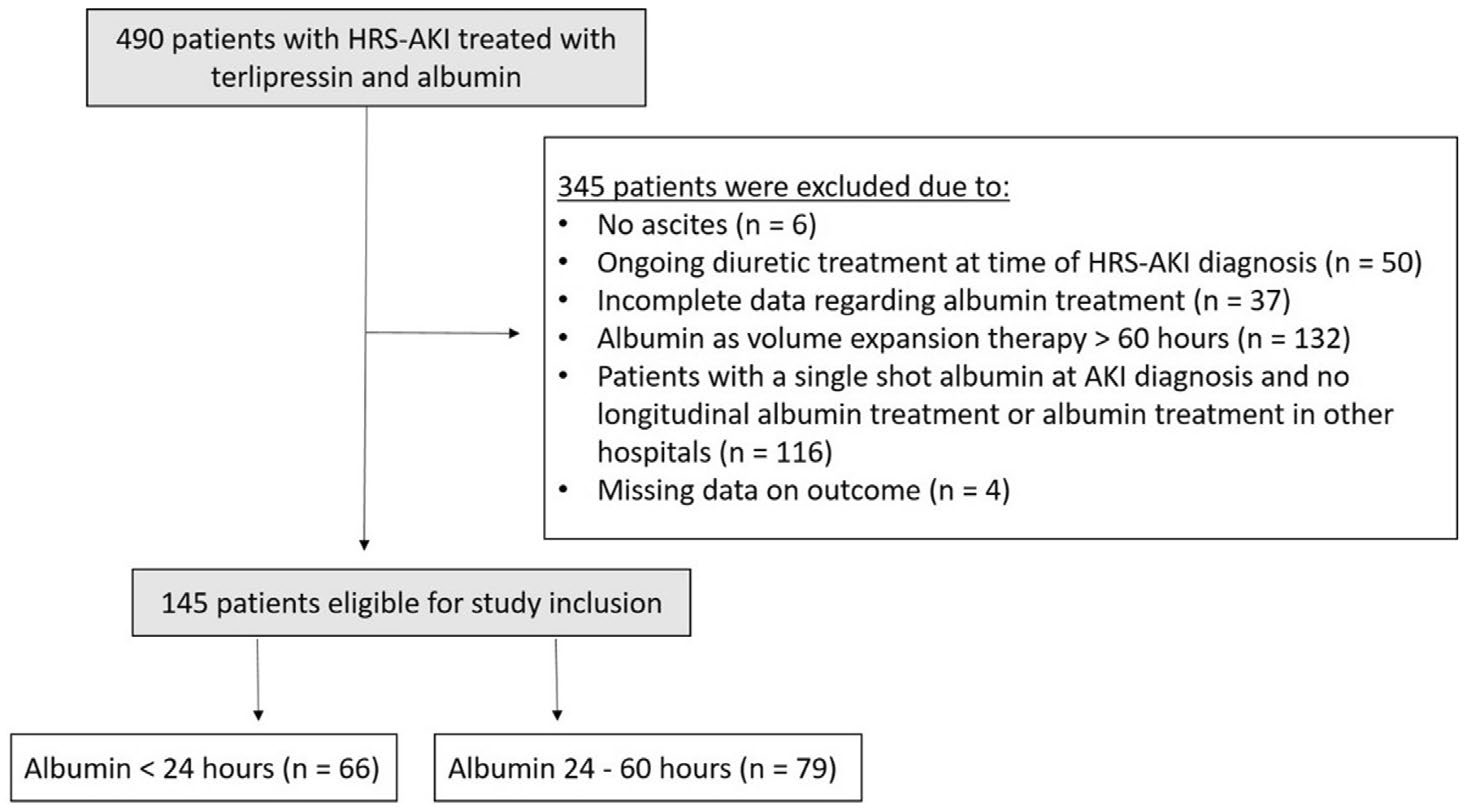
Study flowchart. AKI, acute kidney injury; HD, haemodialysis; HRS‐AKI, hepatorenal syndrome—acute kidney injury.

### 2.2 Definition of Acute Kidney Injury (AKI) and Hepatorenal Syndrome (HRS‐AKI)

AKI was defined by Kidney Disease Improving Global Outcomes (KDIGO) criteria, which the ICA has endorsed as an increase in SCr of at least 0.3 mg/dL (26.5 μmol/L) within 48 h or a percentage increase in SCr of at least 50% from baseline, which is already known or presumed to have occurred within the prior seven days [12]. When baseline values were unavailable or when AKI was already present upon hospital admission, the lowest SCr value recorded within the previous 3–6 months was used as the baseline. The aforementioned criteria classify AKI into different stages (AKI Stage 1A and B, AKI Stage 2, and AKI Stage 3) [13, 14, 15]. For the present study, AKI Stage 1A and 1B were combined and referred to as Stage 1 for further analysis. HRS‐AKI was diagnosed according to the ICA‐AKI criteria [12].

### 2.3 Definition of Response to Terlipressin and Albumin

Terlipressin treatment was initiated in accordance with the German clinical guidelines, with administration carried out according to the local standard practices [16]. Treatment response to terlipressin and albumin was defined according to currently applicable guidelines (European Association for the Study of the Liver (EASL), ICA, and American Association for the Study of Liver Diseases (AASLD)). A complete response was defined as SCr decreasing back to within 0.3 mg/dL of the baseline value, and a partial treatment response was defined as a decrease in SCr that results in an improvement of at least one AKI stage without meeting the criteria for complete treatment response [10, 17]. For the purpose of the present analysis, treatment response was analysed as a dichotomous outcome, with both complete and partial response categorized as “any response”.

### 2.4 Follow‐Up Evaluations

All patients were followed via electronic chart review for up to 12 months after the HRS‐AKI episode for the initiation of HD as well as LT, or death. The start of follow‐up was defined as the date of HRS‐AKI diagnosis and initiation of terlipressin therapy. Thus, all patients entered the risk set at the time of treatment initiation, and no observation time prior to terlipressin exposure was included in the analysis.

### 2.5 Ethics

This study was conducted in accordance with the ethical guidelines of the 1975 Declaration of Helsinki and its later amendments. Ethics committee approval for the collection of the retrospective data with waiver of informed consent was first obtained in the Jena University Hospital (Reg. N° 2023‐3196‐Daten) and subsequently obtained by all participating centres (Freiburg: 24‐1020‐S1‐AV, Mainz: 2024‐17 457, Münster: 2024‐066‐b‐S, Aachen: 24‐069; Berlin: EA1/276/24, Bonn: 2024‐268‐BO; Frankfurt: 20‐707 and 20‐653, Halle: 2024‐095, Munich: 22‐0171, Hannover: 3188‐2016 and 7935_BO_K_2018, Essen: 24‐11 971‐BO). This secondary analysis is part of the study registered at Clinicaltrials.gov (NCT06161766).

### 2.6 Statistical Analyses

All data were analysed using R Software Version 4.4.2 (R Core Team, 2024). Continuous variables were reported as medians with interquartile ranges, and categorical variables were summarized as frequencies and percentages. Comparisons between groups were made using a Man‐Whitney *U* test, unpaired *t*‐test or a chi square or Fisher’s exact test as appropriate. Response to treatment was explored by calculating the cumulative incidence function (CIF), considering death and LT (for response assessment) as competing events. Cumulative incidence functions for complete response and for any response were estimated in a competing‐risks framework. To minimize bias, a propensity score matching approach was also applied. Propensity scores were calculated for individual patients of both albumin duration groups (< 24 h vs. 24–60 h) using the following covariates: age, sex, MELD score, aetiology of liver disease, chronic kidney disease, ACLF, AKI stage, and INR. Matching was performed using nearest neighbour matching (1:1 ratio) with a calliper of 0.2 of the standard deviation of the logit of the propensity score. Patients without suitable matches were excluded from the matched cohort. Covariate balance between groups before and after matching was assessed by comparing standardized mean differences (SMDs) and visualized using jitter and histogram plots of propensity scores. Balance was considered acceptable when SMDs were < 0.1. Comparisons between groups were conducted with Gray's test. A series of multivariable models was constructed to assess the potential associations between albumin treatment duration, adjusted for other relevant risk factors, and HD/LT‐free survival. In all multivariable models, we included albumin treatment duration, regardless of the results of the univariable analyses. First, we built models that included the Model of End‐stage Liver Disease (MELD) or acute‐on‐chronic liver failure (ACLF) scores and albumin treatment duration. Second, we built a model including all univariable variables with *p* < 0.1. Lastly, we built a model that included only MELD, albumin treatment duration, and laboratory variables with *p* < 0.1 not included in MELD. HD/LT‐free survival outcomes were analysed using the Kaplan–Meier method (and log‐rank test). For all tests, we used a 0.05 level to define statistically relevant deviations from the respective null hypotheses. No adjustments for multiple testing were performed.

## 3 Results

### 3.1 Demographics and Baseline Characteristics

The detailed patient flow‐chart is displayed in Figure 1. The median age of the cohort was 59 years (IQR: 51–65), and 63% were male. The predominant aetiology of liver disease was alcohol‐related liver disease (ALD), accounting for 61% of cases, followed by metabolic dysfunction‐associated steatotic liver disease (MASLD, 11%).

The median MELD score at the start of terlipressin therapy was 25 (IQR: 21–31). Most patients were classified as Child‐Pugh class C (55%), while the remaining 45% were classified as class B. CKD was reported in 32%. At the time of AKI diagnosis, AKI Stage 1 was observed in 41% of patients, AKI Stage 2 in 28%, and AKI Stage 3 in 31%. Treatment‐refractory ascites was diagnosed in 72% of patients. ACLF was present in 67% of patients, and 26% had a history of HRS‐AKI. The total cohort was well balanced between patients who received albumin for < 24 h (46%) and those who received albumin for 24–60 h (54%). During follow‐up, 59 patients (40.7%) achieved a complete response to treatment with terlipressin, 74 patients (51.0%) died, and 22 patients (15.2%) received an LT. A detailed description of the total cohort, as well as a comparison of patients treated with albumin for < 24 h or 24–60 h, is presented in Table 1. The propensity‐matched cohort consisted of 100 patients, with 50 patients in each albumin‐duration group (< 24 h and 24–60 h). Baseline characteristics of this cohort are displayed in Table S1.

**TABLE 1:** Demographics and clinical characteristics of the cohort at the time of study inclusion, and stratified by the duration of albumin therapy.

Variable	*N* = 145	< 24 h *n* = 66	24–60 h *n* = 79	*p*
Age (years) (IQR)	59 (51, 65)	59 (52, 66)	58 (51, 64)	0.5
Sex, *n* (%)				0.076
Male, *n* (%)	92 (63%)	47 (71%)	45 (57%)	
Female, *n* (%)	53 (37%)	19 (29%)	34 (43%)	
BMI (kg/m^2^) *n = 142*	26 (23, 30)	25 (23, 29)	27 (23, 30)	0.6
Aetiology				0.2
ALD, *n* (%)	89 (61%)	36 (55%)	53 (67%)	
Viral, *n* (%)	8 (5.5%)	7 (11%)	1 (1.3%)	
MASLD, *n* (%)	16 (11%)	8 (12%)	8 (10%)	
MetALD, *n* (%)	10 (6.9%)	4 (6.1%)	6 (7.6%)	
Autoimmune, *n* (%)	7 (4.8%)	3 (4.5%)	4 (5.1%)	
Other, *n* (%)	15 (10%)	8 (12%)	7 (8.9%)	
MELD score, (IQR)	25 (21, 31)	25 (21, 31)	27 (22, 31)	0.4
Child Pugh				0.5
A, *n* (%)	0 (0%)	0 (0%)	0 (0%)	
B, *n* (%)	65 (45%)	32 (48%)	33 (42%)	
C, *n* (%)	80 (55%)	34 (52%)	46 (58%)	
CKD, *n* (%)	47 (32%)	24 (36%)	23 (29%)	0.4
History of HD, *n* (%)	6 (4.1%)	4 (6.1%)	2 (2.5%)	0.4
ACLF, *n* (%)	97 (67%)	43 (65%)	54 (68%)	0.7
AKI stage				0.053
Stage 1, *n* (%)	59 (41%)	27 (41%)	32 (41%)	
Stage 2, *n* (%)	41 (28%)	13 (20%)	28 (35%)	
Stage 3, *n* (%)	45 (31%)	26 (39%)	19 (24%)	
Ascites at diagnosis of AKI				0.8
Diuretic responsive, *n* (%)	41 (28%)	18 (27%)	23 (29%)	
Treatment refractory, *n* (%)	104 (72%)	48 (73%)	56 (71%)	
History of OHE, *n* (%)	44 (30%)	21 (32%)	23 (29%)	0.7
History of HRS‐AKI, *n* (%)	38 (26%)	14 (21%)	24 (30%)	0.2
History of variceal bleeding, *n* (%)	22 (15%)	16 (24%)	6 (7.6%)	**0.005**
History of TIPS insertion, *n* (%)	16 (11%)	10 (15%)	6 (7.6%)	0.15
HCC, *n* (%)	15 (10%)	10 (15%)	5 (6.3%)	0.082
Comorbidities				
Arterial hypertension, *n* (%)	51 (35%)	26 (39%)	25 (32%)	0.3
Coronary artery disease, *n* (%)	21 (14%)	7 (11%)	14 (18%)	0.2
Congestive heart disease, *n* (%)	7 (4.8%)	3 (4.5%)	4 (5.1%)	> 0.9
Diabetes mellitus, *n* (%)	41 (28%)	18 (27%)	23 (29%)	0.8
Cumulative Albumin dose prior to terlipressin, g/kg BW (IQR)	0.92 (0.52, 1.33)	0.62 (0.36, 1.07)	1.16 (0.73, 1.77)	**< 0.001**
Cumulative dose of terlipressin, mg (IQR), *n* = 127	16 (7, 27)	15 (7, 27)	19 (7, 27)	0.6
Dose of albumin under treatment with terlipressin, g/kg BW (IQR) *n* = 109	0.38 (0.23, 0.54)	0.39 (0.23, 0.56)	0.37 (0.19, 0.52)	0.6
Peak SCr (mg/dL), (IQR)	3.33 (2.47, 4.51)	3.70 (2.43, 4.83)	3.18 (2.47, 4.10)	0.4
^a^ΔSCr, absolute (mg/dL), (IQR)	−0.70 (−1.60, 0.00)	−0.83 (−1.79, −0.06)	−0.68 (−1.33, 0.33)	0.2
^a^ΔSCr, relative (%), (IQR)	−33 (−54, 0)	−36 (−54, −2)	−31 (−55, 9)	0.7
Laboratory parameters				
Sodium (mmol/L), (IQR), *n* = 140	133.0 (129.0, 136.5)	134.0 (130.0, 137.0)	133.0 (128.0, 136.0)	0.4
Bilirubin (mg/dL), (IQR), *n* = 141	3 (2, 10)	3 (1, 9)	4 (2, 12)	0.2
INR, (IQR)	1.63 (1.36, 1.99)	1.53 (1.34, 1.72)	1.68 (1.39, 2.13)	**0.043**
SCr (mg/dL), (IQR)	2.37 (1.90, 3.49)	2.52 (1.90, 4.08)	2.35 (1.90, 3.12)	0.2
Albumin (g/L), (IQR), *n* = 114	28 (24, 32)	28 (22, 31)	28 (25, 33)	0.7
Haemoglobin (g/dL), (IQR)	8.80 (7.60, 10.50)	8.88 (7.80, 10.60)	8.70 (7.60, 10.20)	0.3
Platelets (per nL), (IQR), *n* = 144	99 (66, 147)	107 (71, 146)	89 (55, 147)	0.3
WBC (per nL), (IQR), *n* = 144	8.0 (5.7, 11.7)	8.1 (5.7, 11.9)	7.8 (5.4, 11.4)	0.6
CRP (mg/L), (IQR), *n* = 132	2.9 (1.7, 5.8)	2.9 (1.8, 6.8)	3.0 (1.5, 5.5)	0.5

*Note:* Data are expressed as medians and interquartile ranges or as frequencies and percentages.

Abbreviations: ACLF, acute‐on‐chronic liver failure; AKI, acute kidney injury; ALD, alcohol‐related liver disease; BMI, body mass index; BW, body weight; CRP, C‐reactive protein; h, hours; HCC, hepatocellular carcinoma; HD, haemodialysis; HRS‐AKI, hepatorenal syndrome–acute kidney injury; INR, International Normalized Ratio; IQR, interquartile range; MASLD, metabolic dysfunction–associated steatotic liver disease; MELD, Model of End‐Stage Liver Disease; MetALD, MASLD with significant alcohol intake; n, number; OHE, overt hepatic encephalopathy; SCr, serum creatinine; TIPS, transjugular intrahepatic portosystemic shunt; WBC, white blood cell count.

^a^ ΔSCr was calculated as the difference between SCr at the time of AKI and SCr at the end of terlipressin treatment. Comparisons between groups were made using a Mann–Whitney *U* test, an unpaired *t*‐test, or a chi squared test as appropriate. *p* values in bold designate statistical significance of < 0.05.

### 3.2 Influence of Albumin Treatment Duration on Response to Terlipressin Therapy

Cumulative incidence plots showed no differences in response to terlipressin therapy between albumin treatment groups (< 24 h vs. 24–60 h), neither for any response (complete or partial response) (*p* = 0.7; Figure 2A) nor complete response alone (*p* = 0.9; Figure S1A). Further results of univariable Fine and Gray regression analyses for any response are displayed in Table S2. Univariable regression analyses for complete treatment response are displayed in Table S3. To identify variables independently associated with the response to terlipressin therapy, we conducted various multivariable Fine and Gray models. Again, albumin treatment duration was not associated with the composite endpoint in all three models (Table 2).

**FIGURE 2:**
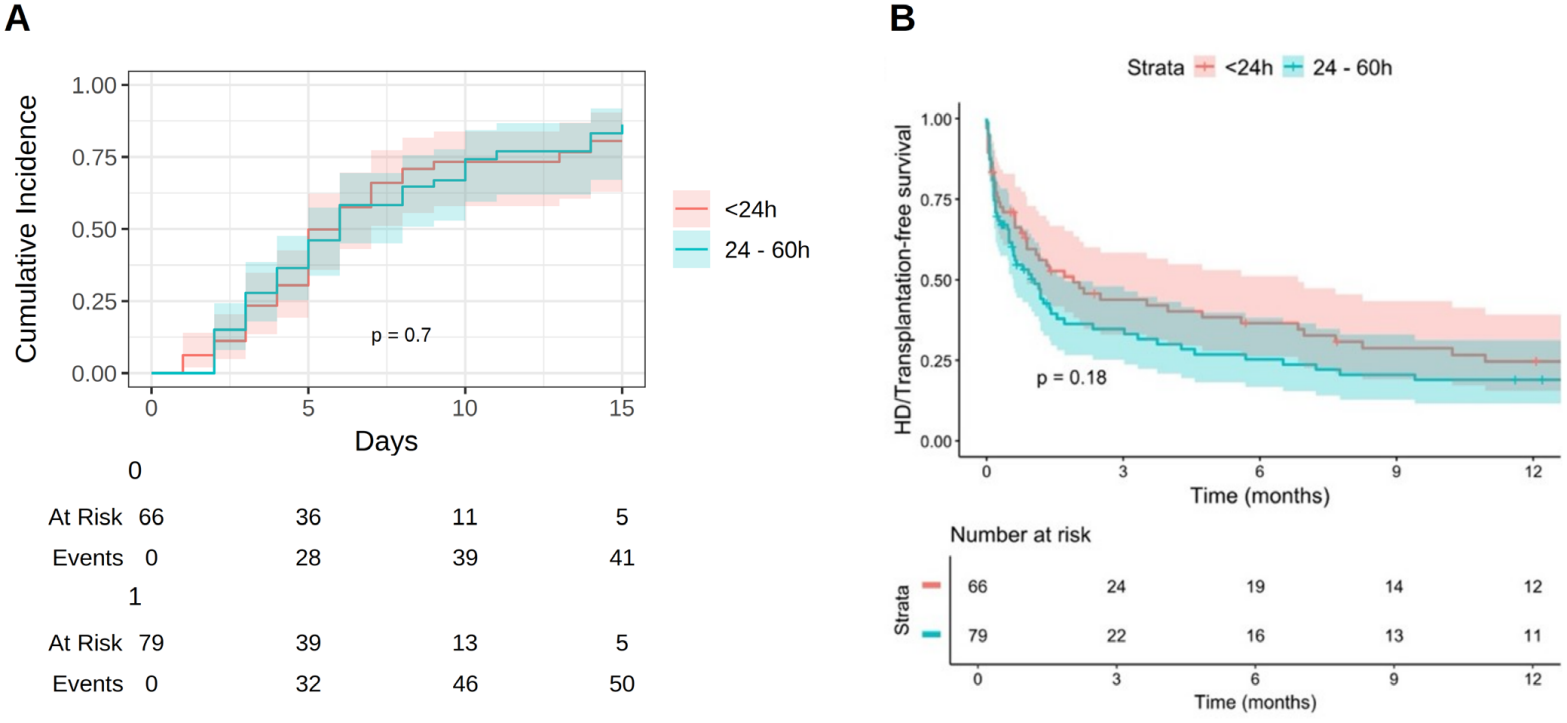
Cumulative incidence of any response to terlipressin therapy (A) and HD‐ and LT‐free survival (B) according to albumin treatment duration (< 24 h vs. 24–60 h). The cumulative incidence of complete or partial response to terlipressin treatment did not differ significantly between groups (A: *p =* 0.7, Gray's test). Kaplan–Meier curve showing the incidence of HD/death/liver transplantation (B, *p* = 0.18, log‐rank test). HD, haemodialysis; LT, liver transplantation.

**TABLE 2:** Multivariable^a^ Fine and Gray regression analysis for identifying variables associated with any response to terlipressin therapy.

Variable	sHR (95% CI)	*p*
Model 1		
MELD score	0.97 (0.94–1.00)	0.077
Albumin duration (< 24 h vs. 24–60 h)	0.96 (0.67–1.40)	0.8
Model 2		
ACLF (yes vs. no)	0.76 (0.52–1.09)	0.13
Albumin duration (< 24 h vs. 24–60 h)	0.93 (0.65–1.33)	0.7
Model 3		
MELD score	0.98 (0.94–1.01)	0.20
Albumin duration (< 24 h vs. 24–60 h)	1.18 (0.80–1.74)	0.4
Congestive heart disease (yes vs. no)	1.89 (0.87–4.08)	0.11
History of variceal bleeding	1.82 (1.05–3.14)	0.033
Haemoglobin	1.11 (1.02–1.21)	0.013
Platelets	1.0 (1.0–1.01)	0.076

Abbreviations: ACLF, acute‐on‐chronic liver failure; h, hours; MELD, Model of End‐Stage Liver Disease; n, number; SCr, serum creatinine; sHR, subdistribution hazard ratio.

^a^ Variables with *p < *0.10 in univariable analysis and albumin treatment duration were included in the multivariable model.

### 3.3 Influence of Albumin Treatment Duration on Prognosis

In Kaplan–Meier analyses, duration of albumin treatment was not associated with HD−/LT‐free survival (median survival time: 1.9 months [95% CI, 0.89–6.8] vs. 1.1 months [95% CI, 0.59–1.7], *p* = 0.18, Figure 2B) nor LT‐free survival (median survival time: 2.1 months [95% CI, 1.2–7.7] vs. 1.2 months [95% CI, 0.79–3.7], *p* = 0.27), respectively (Figure S1B). Results of univariable Cox regression analyses for predictors of HD−/LT‐free survival are displayed in Table S4.

In separate multivariable Cox regression models, albumin treatment duration was not associated with the composite endpoint of HD−/LT‐free survival, whereas the MELD score emerged as an independent predictor of HD‐ and LT‐free survival in Model 1 (Table 3). Multivariable Cox regression analysis for LT‐free survival confirmed these results (Table S5).

**TABLE 3:** Multivariable^a^ Cox regression analysis for HD−/LT‐free survival.

Variable	HR (95% CI)	*p*
Model 1		
MELD	1.03 (1.01–1.06)	0.012
Albumin duration (< 24 h vs. 24–60 h)	1.27 (0.87–1.86)	0.2
Model 2		
ACLF (yes vs. no)	1.12 (0.75–1.68)	0.6
Albumin duration (< 24 h vs. 24–60 h)	1.31 (0.89–1.91)	0.2
Model 3		
MELD	1.01 (0.97–1.05)	0.6
Albumin duration (< 24 h vs. 24–60 h)	1.56 (0.98–2.48)	0.063
Aetiology (ref: ALD)		
Viral	2.03 (0.84–4.93)	0.12
MASLD	1.86 (0.90–3.86)	0.094
MetALD	1.55 (0.68–3.53)	0.3
Autoimmune	2.60 (0.84–8.03)	0.10
Other	4.18 (1.99–8.80)	< 0.001
Treatment refractory ascites at diagnosis (yes vs. no)	1.03 (0.62–1.72)	0.9
Sodium	1.00 (0.96–1.04)	> 0.9
Albumin	0.98 (0.94–1.02)	0.4
Platelets	1.00 (1.00–1.00)	0.3
Model 4		
ACLF stage		
1a	0.93 (0.57–1.52)	0.8
1b	1.22 (0.70–2.15)	0.5
2	1.66 (0.91–3.04)	0.1
3	1.27 (0.56–2.92)	0.6
AKI stage		
2	0.83 (0.52–1.32)	0.4
3	0.70 (0.45–1.19)	0.2
Albumin duration (< 24 h vs. 24–60 h)	1.26 (0.85–1.88)	0.2

Abbreviations: ACLF, acute‐on‐chronic liver failure; MASLD, metabolic dysfunction–associated steatotic liver disease; MetALD, MASLD with significant alcohol intake; MELD, Model of End‐Stage Liver Disease.

^a^ Adjusted for MELD, albumin duration, aetiology, ascites at diagnosis, sodium, albumin, and platelets. Model 1 and 2 include MELD or ACLF together with albumin treatment duration. Model 3 is the fully adjusted model including MELD, albumin duration, aetiology of liver disease, ascites at diagnosis, serum sodium, albumin, and platelet count. Model 4 includes clinically relevant variables as ACLF stage, AKI stage, and albumin duration. Hazard ratios (HR) with 95% confidence intervals (CI) are provided.

### 3.4 Complete Response and HD/LT‐Free Survival in a Subgroup of the Cohort After Propensity Score Matching

To further validate the aforementioned results, we conducted a thorough propensity score matching. After matching (criteria mentioned in the methods section) a cohort of 100 patients (50 per group: albumin for < 24 h and for 24–60 h) remained. In accordance with the results of the total cohort, there was no impact of albumin treatment duration neither on the cumulative incidence of any (*p* = 0.4) or complete response (*p* = 0.8) to terlipressin treatment, nor on HD−/LT‐free (*p* = 0.31) or LT‐free survival (*p* = 0.34) (Figures S2 and S3).

## 4 Discussion

The duration of volume expansion therapy for the diagnosis of HRS‐AKI in patients with cirrhosis remains a subject of considerable debate. Despite the ongoing controversy, data on the impact of albumin duration on subsequent treatment response to terlipressin and on prognosis remain scarce [7, 18]. Thus, determining the optimal duration of albumin administration involves a careful balance between minimizing the risk of overtreatment and ensuring a timely diagnosis of HRS‐AKI. Our current study demonstrates that albumin treatment duration (< 24 h vs. 24–60 h) does not correlate with the likelihood of response to terlipressin in real‐world patients with HRS‐AKI. Additionally, we found no association of albumin treatment duration on HD‐ and LT‐free survival. Recent observational studies showed that a significant proportion of patients respond to volume expansion therapy with albumin beyond the first 24 h, suggesting that shortening the duration of albumin treatment may lead to overtreatment with terlipressin [6, 7, 8]. The present findings further suggest that a delay in initiating terlipressin beyond 24 h does not affect response to terlipressin and long‐term outcomes in this real‐world cohort of patients with HRS‐AKI. Additionally, we validated all our findings by applying a thorough matching.

In recent data from the ICA‐GLOBAL AKI study, a large international prospective observational study, half of the patients who received terlipressin did not meet the HRS‐AKI criteria [19, 20]. In this geographically diverse cohort of patients hospitalized for decompensated cirrhosis and AKI from 65 centers, HRS–AKI represented only a minority of AKI phenotypes (17.4%), with hypovolemia being by far the most common cause (> 50%) [20]. In our current study, which also relies on a multicenter real‐world cohort, we cannot fully exclude that some patients suffered from hypovolemic AKI, especially in the group of patients with albumin treatment < 24 h. Importantly, the findings of our current study can be interpreted in two ways: on the one hand, extending albumin treatment beyond 24 h did not correlate with impaired response to terlipressin treatment; on the other hand, earlier initiation of terlipressin treatment does not worsen prognosis. Indeed, the latter hypothesis would be supported by a prior single‐center randomized trial in patients with ACLF‐associated AKI (AKI Stage 2–3) suggesting that earlier initiation of terlipressin after only 12 h of albumin improves AKI reversal and short‐term survival [5]. Taking this into account, our study's findings have relevant clinical implications. Using the prior recommendation of 48 h of albumin treatment for the biggest subset of patients might avoid unnecessary treatment with terlipressin without impairing treatment effects. This is further supported by recent data showing that up to 40% of patients with AKI stage > 1B who respond to albumin at 48 h do not achieve early response at 24 h [6, 7, 8]. Furthermore, the application of the EASL AKI management algorithm has been associated with very good response rates and does not appear to significantly delay initiation of terlipressin in patients with HRS‐AKI [8]. This is clinically important, given that the results of the CONFIRM trial and subsequent analyses indicate that terlipressin does not confer a clear survival advantage [21, 22].

Furthermore, clinicians must balance the potential renal benefits against a well‐recognized spectrum of adverse effects, including ischemic events, diarrhoea, and respiratory failure. Consequently, early evaluation for liver transplantation should be pursued in parallel, rather than relying solely on pharmacologic therapy for HRS‐AKI. Moreover, terlipressin remains a costly medication in many healthcare systems. In settings where financial resources are limited, the decision to initiate terlipressin becomes even more nuanced, and the risk of terlipressin overtreatment may not only be clinically unfavourable but also economically inefficient, given the cost of terlipressin and the need for intensive monitoring and management of adverse events, especially in patients unlikely to respond [20, 21, 23, 24].

This study has limitations that have to be acknowledged. First, this is a real‐world study, not a randomized controlled trial. Therefore, treatment duration and dosing were at the discretion of the treating physician at the respective center. Consequently, most patients did not receive the guideline‐recommended dose of 1 g albumin per kg body weight per day. Second, no control group treated with a saline solution was available. Therefore, we are unable to analyse the influence of albumin treatment compared to other treatment modalities on terlipressin response. Third, due to its retrospective nature, our analysis does not allow for definitive conclusions regarding potential adverse effects of albumin and terlipressin, such as pulmonary edema or hypotension, as this information was not collected with a granularity of a randomized controlled trial, and the sample size was too small to draw sufficient conclusions. However, recently, it has been shown that respiratory failure is common in this setting but does not appear to be related to the amount of albumin administered [19]. In general, volume expansion for 48 h should always be carefully monitored, especially in non‐responders. Nevertheless, as all patients in our cohort ultimately required terlipressin, they likely represented a population of non‐responders to plasma volume expansion. In this selected cohort, the duration of albumin administration before terlipressin initiation was not associated with mortality. These findings provide additional context to the recent analysis by Piano et al., which reported worse outcomes with greater overall fluid exposure (including crystalloids) among non‐responders [18]. While our study does not address the safety of higher fluid volumes, it suggests that, within a population of albumin non‐responders treated with terlipressin, prolonging albumin administration before terlipressin initiation may not negatively influence patient survival. Thus, albumin volume expansion therapy should be individualized according to the patient's volume status, although the optimal method for assessing volume status in patients with cirrhosis remains uncertain.

In conclusion, this study provides real‐world evidence to inform current clinical practice by demonstrating that albumin treatment duration, whether shorter or longer than 24 h, had no significant impact on terlipressin treatment response. This supports individualized, tailored management strategies but also indicates that expanding albumin treatment beyond 24 h might be reasonable and can help avoid overtreatment with terlipressin.

## Author Contributions

Performed research: all authors. Contributed to acquisition of data: all authors. Designed the experiments and analysed the data: Eva Maria Schleicher, Frank Erhard Uschner, Dominik Bettinger, Cristina Ripoll. Contributed reagents/materials/analysis tools: NA. Wrote the paper: Eva Maria Schleicher, Christian Labenz, Frank Erhard Uschner, Cristina Ripoll, Dominik Bettinger. Critical revision of the draft: all authors. Statistical analysis: Eva Maria Schleicher, Christian Labenz. All authors approved the final version of the manuscript and the authorship list. Guarantor of the article: Dominik Bettinger.

## Funding

The authors have nothing to report.

## Conflicts of Interest

Eva Maria Schleicher: received lecture fees from the Falk Foundation, travel grants from Boehringer Ingelheim, AbbVie, Gilead, Merz therapeutics, Advitos. Christian Labenz: received lecture fees from CSL Behring. Frank Erhard Uschner: receives lecture fees from AstraZeneca, Gilead, AbbVie and the Falk Foundation and travel grants from AbbVie. Julian Pohl received travel grants from Ipsen and AbbVie. Moritz Passenberg: no conflicts of interest. Michael Praktiknjo received speaker fees from Gore, AbbVie, Falk, MicroTech, Cook. Michael Praktiknjo received consultant fees from Gore, AstraZeneca, Roche, Cook, Ipsen. Michael Praktiknjo received travel grants from Univar, Gilead, Cook, Ipsen. Marcus Maximilian Mücke received speaker fees from AbbVie, BioMarin, Ipsen, travel grants from AbbVie and Ipsen. Henrik Karbannek: no conflict of interests. Nina Böhling: no conflict of interests. Karsten Große: no conflict of interests. Marlene Reincke received lecture fees from W. L. Gore & Associates GmbH and the Falk Foundation and a travel grant from W. L. Gore & Associates GmbH. Sarah Schütte: no conflict of interests. Paul Jamme: no conflict of interests. Julian Cardinal von Widdern received travel grants from W. L. Gore & Associates GmBH, Sanofi Regeneron and AbbVie. Research Grants: Advanced Clinician Scientist Programme (ACCENT funding code 01EO2107) sponsored by the German Federal Ministry of Education and Research (BMBF), Ernst und Berta Grimmke Foundation No. 6/23. Christoph Welsch: no conflict of interests. Jonel Trebicka received speaking and/or consulting fees from Versantis, Gore, Boehringer‐Ingelheim, Falk, Grifols, Genfit and CSL Behring. Cornelius Engelmann: Lecture fees from Falk Foundation, Ipsen, Gilead, Doctorflix, Pfizer. Advisory board: Boehringer Ingelheim, Ipsen, Alfasigma. Travel grants: Ipsen. Shareholder of Yakrit Ltd. Jassin Rashidi‐Alavijeh received speaker and consultant fees from AbbVie, Ipsen, Sobi, Falk, Chiesi, Alexion. Travel grants from Ipsen, AbbVie, Gilead and Merz. Cristina Ripoll: Lecture fees from W. L. Gore & Associates GmbH, the Falk Foundation and Bristol‐Myers‐Squibb. Advisory Board: Boehringer Ingelheim. Research grants: German Research Foundation DFG (Deutsche Forschung Gemeinschaft) Project number 431667134 and European Union Horizon 2020 research and innovation programme under grant agreement 101136299 ARTEMIS. Dominik Bettinger: received lecture fees from W. L. Gore & Associates GmbH and the Falk Foundation and a travel grant from W. L. Gore & Associates GmbH. Research grants: Dr. Rolf M. Schwiete‐Stiftung (project number: 2023‐019), German Research Foundation (DFG, project number: 529465923).

## Supporting information


**Figure S1:** Cumulative incidence of complete response (A) and LT‐free survival (B) according to albumin treatment duration (< 24 h vs. 24–60 h).
**Table S1:** Univariable Fine and Gray regression analysis for identifying variables associated with any response.
**Table S2:** Univariable Fine and Gray regression analysis for identifying variables associated with complete response.
**Table S3:** Univariable Cox regression analysis of variables associated with the composite endpoint of HD, LT or death during the complete follow‐up.
**Table S4:** Multivariable Cox regression analysis for LT‐free survival.
**Table S5:** Comparison of baseline characteristics of the propensity matched cohort stratified by the duration of albumin therapy.
**Figure S2:** Cumulative incidence of any response to terlipressin therapy (A) and HD‐ and LT‐free survival (B) according to albumin treatment duration (< 24 h vs. 24–60 h) after propensity score matching.
**Figure S3:** Cumulative incidence of complete response to terlipressin therapy (A) and LT‐free survival (B) according to albumin treatment duration (< 24 h vs. 24–60 h) after propensity score matching.

## Data Availability

Data are available on reasonable request.

## Appendix A German Cirrhosis Study Group

Johannes Chang^7^, Maximilian Joseph Brol^2^, Tony Bruns^8^, Marko Damm^12^, Jan Hendrik Harten^2^, Christian Jansen^7^, Alexander Queck^5^, Christian Lange^1^, Lauin Mustafa^2^, Benjamin Maasoumy^10^, Andreas Stallmach^6^, Anna Teresa Volk^5^, and Alexander Zipprich^6^.
