# Supplementary material for: Influence of Albumin Treatment Duration on Outcome of Terlipressin Therapy in Patients With HRS‐AKI
Source: Liver Int. 2026 Sep 30;46(11):e70888. doi: 10.1111/liv.70888 (PMC13625807; doi:10.1111/liv.70888)
Supplement: Supplementary file 1 [file LIV-46-0-s001.docx]

**Influence of albumin treatment duration on outcome of terlipressin therapy in patients with hepatorenal syndrome-acute kidney injury**

Eva Maria Schleicher^1*^, Christian Labenz^1*^, Frank Erhard Uschner^2*^, Julian Pohl^3^, Moritz Passenberg^4^, Michael Praktiknjo^2^, Marcus Maximilian Mücke^5^, Henrik Karbannek^6^, Nina Böhling^7^, Karsten Große^8^, Marlene Reincke^9^, Sarah Schütte^10^, Paul Jamme^11^, Julian Cardinal von Widdern^12^, Christoph Welsch^5^, Jonel Trebicka^2^, Cornelius Engelmann^4^, Jassin Rashidi-Alavijeh^3^, Cristina Ripoll^6*^, Dominik Bettinger^9*^, *on behalf of the German cirrhosis study group*

*German cirrhosis study group*: Johannes Chang^7^, Maximilian Joseph Brol^2^, Tony Bruns^8^, Marko Damm^12^, Jan Hendrik Harten^2^, Christian Jansen^7^, Alexander Queck^5^, Christian Lange^11^, Lauin Mustafa^2^, Benjamin Maasoumy^10^, Andreas Stallmach^6^, Anna Teresa Volk^5^ and Alexander Zipprich^6^

^1^Department of Internal Medicine I, University Medical Center of the Johannes Gutenberg-University, Mainz, Germany

^2^Department of Internal Medicine B, University Hospital Münster, Münster, Germany

^3^Department of Hepatology and Gastroenterology, Charité-Universitätsmedizin Berlin, Berlin, Germany

^4^Department of Gastroenterology, Hepatology and Transplant Medicine, University of Duisburg-Essen, Essen, Germany

^5^Medical Clinic 1, University Hospital, Goethe-University Frankfurt, Frankfurt am Main, Germany

^6^Department of Internal Medicine IV, Jena University Hospital, Jena, Germany

^7^Department of Internal Medicine I, Bonn University Hospital, Bonn, Germany

^8^Department of Internal Medicine III, University Hospital RWTH Aachen, Aachen, Germany

^9^Department of Medicine II, Medical Center University of Freiburg, Freiburg, Germany

^10^Department of Gastroenterology, Hepatology, Infectious Diseases and Endocrinology, Hannover Medical School, Hannover, Germany

^11^Department of Internal Medicine II, LMU University Hospital Munich, Munich, Germany

^12^Department of Internal Medicine I, University Hospital Halle, Martin-Luther-University Halle-Wittenberg, Halle, Germany

*authors share authorship

**Supplementary data**

**Supplementary Figure 1**

**Supplementary Figure 1: Cumulative incidence of complete response (A) and LT-free survival (B) according to albumin treatment duration (<24 hours vs. 24–60 hours)**

The cumulative incidence of complete response to terlipressin treatment did not differ significantly between groups (A: p = 0.9, Gray’s test). Kaplan-Meier curves showing the incidence of liver transplantation-free survival (B, p = 0.27) stratified by albumin treatment duration.

*LT - liver transplantation*

| **Supplementary table 1** | | | |
| --- | --- | --- | --- |
| **Variable** | **<24 hours**  **n = 50** | **24 - 60 hour**  **n = 50** | **p-value^2^** |
| Age (years) (IQR) | 59 (53, 65) | 59 (52, 65) | >0.9 |
| Sex, n (%) |  |  | 0.8 |
| Male, n (%) | 33 (66%) | 32 (64%) |  |
| female, n (%) | 17 (34%) | 18 (36%) |  |
| BMI (kg/m^2^) *n=142* | 25 (23, 29) | 26 (23, 30) | 0.5 |
| Etiology |  |  | 0.9 |
| ALD, n (%) | 31 (62%) | 29 (58%) |  |
| Viral, n (%) | 1 (2.0%) | 1 (2.0%) |  |
| MASLD, n (%) | 6 (12%) | 7 (14%) |  |
| MetALD, n (%) | 2 (4.0%) | 5 (10%) |  |
| Autoimmune, n (%) | 3 (6.0%) | 3 (6.0%) |  |
| Other, n (%) | 7 (14%) | 5 (10%) |  |
| MELD score, (IQR) | 25 (21, 33) | 25 (21, 31) | 0.8 |
| Child Pugh |  |  | 0.8 |
| A, n (%) | 0 (0%) | 0 (0%) |  |
| B, n (%) | 22 (44%) | 24 (48%) |  |
| C, n (%) | 28 (56%) | 26 (52%) |  |
| CKD, n (%) | 16 (32%) | 20 (40%) | 0.4 |
| ACLF, n (%) | 33 (66%) | 35 (70%) | 0.7 |
| AKI stage, n=259 |  |  | 0.8 |
| Stage 1, n (%) | 20 (40%) | 22 (44%) |  |
| Stage 2, n (%) | 13 (26%) | 14 (28%) |  |
| Stage 3, n (%) | 17 (34%) | 14 (28%) |  |
| Ascites at diagnosis of AKI |  |  | 0.8 |
| diuretic responsive, n (%) | 16 (32%) | 15 (30%) |  |
| treatment refractory, n (%) | 34 (68%) | 35 (70%) |  |
| History of OHE, n (%) | 12 (24%) | 17 (34%) | 0.3 |
| History of HRS-AKI, n (%) | 9 (18%) | 16 (32%) | 0.11 |
| History of variceal bleeding, n (%) | 10 (20%) | 4 (8.0%) | 0.084 |
| History of TIPS insertion, n (%) | 8 (16%) | 4 (8.0%) | 0.2 |
| HCC, n (%) | 6 (12%) | 4 (8.0%) | 0.5 |
| Comorbidities:  Arterial hypertension, n (%) | 17 (34%) | 19 (38%) | 0.7 |
| Coronary artery disease, n (%) | 5 (10%) | 13 (26%) | 0.037 |
| Congestive heart disease, n (%) | 0 (0%) | 1 (2.0%) | >0.9 |
| Non-metastatic tumor disease, n (%) | 9 (18%) | 7 (14%) | 0.6 |
| Diabetes mellitus, n (%) | 12 (24%) | 19 (38%) | 0.13 |
| History of HD, n (%) | 3 (6.0%) | 2 (4.0%) | >0.9 |
| Peak SCr (mg/dL), (IQR) | 3.72 (2.49, 4.83) | 3.22 (2.50, 4.31) | 0.5 |
| ΔSCr, absolute (mg/dL), (IQR) | -0.69 (-1.79, 0.15) | -0.79 (-1.53, -0.15) | >0.9 |
| ΔSCr, relative (%), (IQR) | -36 (-54, 7) | -36 (-55, -12) | 0.6 |
| Albumin dose, mg (IQR) | 0.64 (0.43, 1.13) | 1.18 (0.74, 2.01) | **0.003** |
| Dose of terlipressin, mg (IQR), n=127 | 16 (7, 25) | 20 (7, 28) | 0.5 |
| Dose of albumin under treatment with terlipressin, mg (IQR) n=109 | 0.36 (0.22, 0.54) | 0.39 (0.24, 0.55) | 0.6 |
| Laboratory parameters:  Sodium (mmol/L), (IQR), n=140 | 132.5 (129.0, 136.0) | 133.5 (129.0, 137.0) | 0.7 |
| Bilirubin (mg/dL), (IQR), n=141 | 4 (2, 12) | 3 (2, 9) | 0.7 |
| INR, (IQR) | 1.64 (1.39, 1.89) | 1.65 (1.37, 2.00) | 0.7 |
| SCr (mg/dL), (IQR) | 2.34 (1.90, 3.87) | 2.55 (2.04, 3.19) | 0.9 |
| Albumin (g/L), (IQR), n=114 | 27.5 (22.0, 30.3) | 29.0 (25.0, 33.0) | 0.3 |
| Hemoglobin (g/dL), (IQR) | 9.25 (7.80, 10.60) | 8.40 (7.60, 10.10) | 0.2 |
| Platelets (per nL), (IQR), n=144 | 117 (71, 152) | 90 (58, 147) | 0.3 |
| WBC (per nL), (IQR), n=144 | 8.2 (6.0, 12.3) | 7.2 (5.4, 10.4) | 0.093 |
| CRP (mg/L), (IQR), n=132 | 3.1 (1.8, 7.4) | 3.1 (1.7, 4.8) | 0.6 |

*^1^*Median (Q1, Q3); n (%)

**Supplementary Table 1: Comparison of baseline characteristics of the propensity matched cohort stratified by the duration of albumin therapy.**

Data are expressed as medians and interquartile ranges or as frequencies and percentages. Comparisons between groups were made using a Mann-Whitney U test, an unpaired t-test, or a chisquared test. p values in bold designate statistical significance of <0.05.

*ACLF – acute-on-chronic liver failure, AKI – acute kidney injury, ALD – alcohol-related liver disease, BMI – body mass index, CRP – C-reactive protein, HCC – hepatocellular carcinoma, HD – hemodialysis, HRS-AKI – hepatorenal syndrome–acute kidney injury, INR – International Normalized Ratio, IQR – interquartile range, MASLD – metabolic dysfunction–associated liver disease, metALD – MASLD with significant alcohol intake, MELD – Model of End-Stage Liver Disease, n – number, OHE – overt hepatic encephalopathy, SCr – serum creatinine, TIPS – transjugular intrahepatic portosystemic stent, WBC – white blood cell count.*

| **Supplementary table 2** | | |
| --- | --- | --- |
| **Variable** | **sHR (95 % CI)** | **p-value** |
| Age (years) | 1.00 (0.99 – 1.02) | 0.8 |
| Sex (female vs. male) | 0.95 (0.66 – 1.35) | 0.8 |
| BMI (kg/m^2^) | 0.98 (0.95 – 1.01) | 0.2 |
| MASLD | 1.32 (0.81 – 2.15) | 0.3 |
| metALD | 1.12 (0.51 – 2.46) | 0.8 |
| Viral | 0.95 (0.36 – 2.48) | >0.9 |
| Autoimmune | 0.81 (0.42 – 1.55) | 0.5 |
| Other | 0.70 (0.31 – 1.58) | 0.4 |
| MELD score | 0.97 (0.94 – 1.00) | 0.074 |
| Child-Pugh score (C vs. B) | 1.06 (0.73 – 1.54) | 0.8 |
| CKD (yes vs. no) | 0.74 (0.49 – 1.11) | 0.14 |
| History of HD (yes vs. no) | 0.81 (0.30 – 2.16) | 0.7 |
| ACLF (yes vs. no) | 0.76 (0.52 – 1.09) | 0.14 |
| AKI stage |  |  |
| Stage 2 | 1.23 (0.76 – 1.98) | 0.4 |
| Stage 3 | 0.97 (0.63 – 1.50) | 0.9 |
| Ascites at diagnosis (refractory vs. responsive) | 0.83 (0.56 – 1.22) | 0.3 |
| History of OHE (yes vs. no) | 0.90 (0.61 – 1.32) | 0.6 |
| History of HRS-AKI (yes vs. no) | 0.93 (0.60 – 1.42) | 0.7 |
| History of variceal bleeding (yes vs. no) | 1.63 (0.99 – 2.68) | 0.054 |
| History of TIPS insertion (yes vs. no) | 0.92 (0.50 – 1.68) | 0.8 |
| HCC (yes vs. no) | 1.01 (0.55 – 1.85) | >0.9 |
| Comorbidities: (yes vs. no)  Arterial hypertension | 0.85 (0.56 – 1.28) | 0.4 |
| Coronary artery disease | 1.21 (0.72 – 2.04) | 0.5 |
| Congestive heart disease | 2.00 (0.96 – 4.17) | 0.064 |
| Non-metastatic tumor disease | 0.82 (0.45 – 1.48) | 0.5 |
| Diabetes mellitus | 0.86 (0.54 – 1.38) | 0.5 |
| Albumin duration (<24 h vs. 24-60 h) | 0.94 (0.65 – 1.34) | 0.7 |
| Cumulative Albumin dose prior to terlipressin (g/kg BW) | 1.03 (0.79 – 1.34) | 0.8 |
| Cumulative dose of terlipressin (mg) | 0.97 (0.96 – 0.99) | **<0.001** |
| Dose of albumin under treatment with terlipressin (g/kg BW) | 1.03 (0.57 – 1.86) | >0.9 |
| Peak SCr (mg/dL) | 0.77 (0.67 – 0.90) | **<0.001** |
| ΔSCr, absolute (mg/dL) | 0.83 (0.73 – 0.95) | **0.005** |
| Laboratory parameters:  Sodium (mmol/L) | 0.98 (0.95 – 1.01) | 0.2 |
| Bilirubin (mg/dL) | 1.00 (0.98 – 1.02) | 0.7 |
| INR | 0.84 (0.65 – 1.09) | 0.2 |
| SCr (mg/dL) | 0.86 (0.74 – 1.01) | 0.062 |
| Albumin (g/L) | 1.01 (0.98 – 1.05) | 0.4 |
| Hemoglobin (g/dL) | 1.10 (1.01 – 1.19) | **0.023** |
| Platelets (per nL) | 1.00 (1.00 – 1.01) | **0.004** |
| WBC (per nL) | 1.00 (0.98 – 1.02) | 0.8 |
| CRP (mg/L) | 0.96 (0.92 – 1.01) | 0.11 |

**Supplementary Table 2: Univariable Fine and Gray regression analysis for identifying variables associated with any response.**

Data are expressed as subdistribution hazard ratios (sHRs) with 95% confidence intervals (CIs) and corresponding p-values derived from univariable Fine and Gray regression analysis. ΔSCr was calculated as the difference between SCr at the time of AKI and SCr at the end of terlipressin treatment. P values in bold designate statistical significance of <0.05

*ACLF – acute-on-chronic liver failure, AKI – acute kidney injury, BMI – body mass index, BW – body weight, CRP – C-reactive protein, HCC – hepatocellular carcinoma, HD – hemodialysis, HRS-AKI – hepatorenal syndrome–acute kidney injury, INR – International Normalized Ratio, MASLD – metabolic dysfunction–associated liver disease, metALD – MASLD with significant alcohol intake, MELD – Model of End-Stage Liver Disease, n – number, OHE – overt hepatic encephalopathy, SCr – serum creatinine, TIPS – transjugular intrahepatic portosystemic shunt, WBC – white blood cell count.*

| **Supplementary table 3** | | |
| --- | --- | --- |
| **Variable** | **sHR (95 % CI)** | **p-value** |
| Age (years) | 1.02 (1.00–1.04) | 0.12 |
| Sex (female vs. male) | 0.83 (0.51–1.34) | 0.40 |
| BMI (kg/m^2^) | 0.99 (0.95–1.02) | 0.50 |
| MASLD | 1.11 (0.38–3.21) | 0.90 |
| metALD | 1.88 (1.02–3.43) | **0.041** |
| Viral | 1.81 (0.80–4.08) | 0.20 |
| Autoimmune | 0.82 (0.33–2.03) | 0.70 |
| Other | 0.73 (0.23–2.27) | 0.60 |
| MELD score | 0.95 (0.91–0.99) | **0.023** |
| Child-Pugh score (C vs. B) | 0.97 (0.60–1.59) | >0.90 |
| CKD (yes vs. no) | 0.93 (0.55–1.59) | 0.80 |
| History of HD (yes vs. no) | 1.41 (0.53–3.79) | 0.50 |
| ACLF (yes vs. no) | 0.60 (0.36–0.99) | **0.045** |
| AKI stage |  |  |
| Stage 2 | 0.87 (0.46–1.63) | 0.70 |
| Stage 3 | 0.56 (0.31–1.00) | 0.052 |
| Ascites at diagnosis (refractory vs. responsive) | 0.83 (0.49–1.40) | 0.50 |
| History of OHE (yes vs. no) | 1.36 (0.84–2.21) | 0.20 |
| History of HRS-AKI (yes vs. no) | 1.12 (0.66–1.88) | 0.70 |
| History of variceal bleeding (yes vs. no) | 2.32 (1.30–4.16) | **0.005** |
| History of TIPS insertion (yes vs. no) | 1.02 (0.42–2.48) | >0.90 |
| HCC (yes vs. no) | 1.68 (0.86–3.27) | 0.13 |
| Comorbidities: (yes vs. no)  Arterial hypertension | 1.07 (0.64–1.79) | 0.80 |
| Coronary artery disease | 1.46 (0.72–2.99) | 0.30 |
| Congestive heart disease | 0.64 (0.14–2.92) | 0.60 |
| Non-metastatic tumor disease | 1.02 (0.49–2.10) | >0.90 |
| Diabetes mellitus | 0.86 (0.46–1.59) | 0.60 |
| Albumin duration (<24 h vs. 24-60 h) | 0.95 (0.58–1.56) | 0.90 |
| Cumulative Albumin dose prior to terlipressin (g/kg BW) | 0.95 (0.68–1.33) | 0.80 |
| Cumulative dose of terlipressin (mg) | 0.97 (0.95–0.99) | **0.005** |
| Dose of albumin under treatment with terlipressin (g/kg BW) n=109 | 1.14 (0.65–2.00) | 0.70 |
| Peak SCr (mg/dL) | 0.66 (0.51–0.86) | **0.002** |
| ΔSCr, absolute (mg/dL) | 0.77 (0.65–0.90) | **0.001** |
| Laboratory parameters:  Sodium (mmol/L) | 0.97 (0.93–1.01) | 0.20 |
| Bilirubin (mg/dL) | 0.97 (0.93–1.01) | 0.20 |
| INR | 0.82 (0.52–1.27) | 0.40 |
| SCr (mg/dL) | 0.81 (0.65–1.00) | **0.047** |
| Albumin (g/L) | 1.04 (1.00–1.09) | **0.040** |
| Hemoglobin (g/dL) | 1.13 (1.00–1.27) | **0.051** |
| Platelets (per nL) | 1.00 (1.00–1.01) | **0.032** |
| WBC (per nL) | 1.00 (0.97–1.03) | 0.80 |
| CRP (mg/L) | 0.97 (0.91–1.03) | 0.30 |

**Supplementary Table 3: Univariable Fine and Gray regression analysis for identifying variables associated with complete response.**

Data are expressed as subdistribution hazard ratios (sHRs) with 95% confidence intervals (CIs) and corresponding p-values derived from univariable Fine and Gray regression analysis. ΔSCr was calculated as the difference between SCr at the time of AKI and SCr at the end of terlipressin treatment. P values in bold designate statistical significance of <0.05

*ACLF – acute-on-chronic liver failure, AKI – acute kidney injury, BMI – body mass index, BW – body weight, CRP – C-reactive protein, HCC – hepatocellular carcinoma, HD – hemodialysis, HRS-AKI – hepatorenal syndrome–acute kidney injury, INR – International Normalized Ratio, MASLD – metabolic dysfunction–associated liver disease, metALD – MASLD with significant alcohol intake, MELD – Model of End-Stage Liver Disease, n – number, OHE – overt hepatic encephalopathy, SCr – serum creatinine, TIPS – transjugular intrahepatic portosystemic shunt, WBC – white blood cell count.*

| **Supplementary table 4** | | |
| --- | --- | --- |
| **Variable** | **HR (95 % CI)** | **p-value** |
| Age (years) | 1.01 (0.99–1.03) | 0.20 |
| Sex (female vs. male) | 1.21 (0.82–1.80) | 0.30 |
| BMI (kg/m^2^) | 1.02 (0.99–1.05) | 0.30 |
| MASLD | 1.62 (0.77–3.41) | 0.20 |
| metALD | 1.30 (0.70–2.43) | 0.40 |
| Viral | 1.65 (0.78–3.47) | 0.20 |
| Autoimmune | 2.80 (1.27–6.21) | **0.011** |
| Other | 2.17 (1.18–3.97) | **0.012** |
| MELD score | 1.04 (1.01–1.06) | **0.010** |
| Child-Pugh score (C vs. B) | 1.74 (1.18–2.58) | **0.006** |
| CKD (yes vs. no) | 1.10 (0.74–1.65) | 0.60 |
| History of HD (yes vs. no) | 1.16 (0.47–2.84) | 0.80 |
| ACLF (yes vs. no) | 1.11 (0.74–1.66) | 0.60 |
| AKI stage |  |  |
| Stage 2 | 0.85 (0.54–1.35) | 0.50 |
| Stage 3 | 0.74 (0.47–1.17) | 0.20 |
| Ascites at diagnosis (refractory vs. responsive) | 0.89 (0.58–1.35) | 0.60 |
| History of OHE (yes vs. no) | 0.87 (0.58–1.32) | 0.50 |
| History of HRS-AKI (yes vs. no) | 1.00 (0.66–1.53) | >0.90 |
| History of variceal bleeding (yes vs. no) | 0.76 (0.44–1.29) | 0.30 |
| History of TIPS insertion (yes vs. no) | 0.70 (0.38–1.31) | 0.30 |
| HCC (yes vs. no) | 1.30 (0.74–2.28) | 0.40 |
| Comorbidities: (yes vs. no)  Arterial hypertension | 0.85 (0.57–1.27) | 0.40 |
| Coronary artery disease | 0.81 (0.47–1.39) | 0.40 |
| Congestive heart disease | 0.78 (0.28–2.12) | 0.60 |
| Non-metastatic tumor disease | 1.15 (0.69–1.94) | 0.60 |
| Diabetes mellitus | 0.98 (0.64–1.49) | >0.90 |
| Albumin duration (<24 h vs 24-60 h) | 1.30 (0.89–1.90) | 0.20 |
| Cumulative Albumin dose prior to terlipressin (g/kg BW) | 0.95 (0.78–1.15) | 0.60 |
| Cumulative dose of terlipressin (mg) | 1.00 (0.99–1.01) | 0.80 |
| Dose of albumin under treatment with terlipressin (g/kg BW) | 0.92 (0.47–1.82) | 0.80 |
| Peak SCr (mg/dL) | 1.10 (0.97–1.25) | 0.20 |
| ΔSCr, absolute (mg/dL) | 1.38 (1.22–1.55) | **<0.001** |
| Laboratory parameters:  Sodium (mmol/L) | 0.99 (0.96–1.03) | 0.60 |
| Bilirubin (mg/dL) | 1.01 (1.00–1.03) | 0.069 |
| INR | 1.34 (1.07–1.67) | **0.010** |
| SCr (mg/dL) | 0.92 (0.78–1.10) | 0.40 |
| Albumin (g/L) | 0.98 (0.94–1.01) | 0.20 |
| Hemoglobin (g/dL) | 0.96 (0.87–1.05) | 0.40 |
| Platelets (per nL) | 1.00 (0.99–1.00) | **0.017** |
| WBC (per nL) | 1.01 (0.98–1.04) | 0.50 |
| CRP (mg/L) | 1.04 (1.00–1.08) | **0.046** |

**Supplementary Table 4: Univariable Cox regression analysis of variables associated with the composite endpoint of HD, LT or death during the complete follow-up.**

Data are expressed as hazard ratios (HRs) with 95% confidence intervals (CIs) and corresponding p-values derived from univariable Cox regression analysis. ΔSCr was calculated as the difference between SCr at the time of AKI and SCr at the end of terlipressin treatment. P values in bold designate statistical significance of <0.05.

*ACLF – acute-on-chronic liver failure, AKI – acute kidney injury,, BMI – body mass index, BW – body weight, CRP – C-reactive protein, HCC – hepatocellular carcinoma, HD – hemodialysis, INR – International Normalized Ratio, MASLD – metabolic dysfunction–associated liver disease, metALD – MASLD with significant alcohol intake, MELD – Model of End-Stage Liver Disease, OHE – overt hepatic encephalopathy, SCr – serum creatinine, TIPS – transjugular intrahepatic portosystemic shunt, WBC – white blood cell count.*

| **Supplementary table 5** | | |
| --- | --- | --- |
| **Variable** | **HR (95%CI)** | **p-value** |
| Model 1 |  |  |
| MELD score | 1.03 (1.00 – 1.06) | **0.035** |
| Albumin duration (<24 h vs. 24-60 h) | 1.23 (0.82 – 1.84) | 0.3 |
| Model 2 |  |  |
| ACLF (yes vs. no) | 0.98 (0.65 – 1.49) | >0.9 |
| Albumin duration (<24 h vs. 24-60 h) | 1.26 (0.84 – 1.88) | 0.3 |
| Model 3: Adjusted multivariable model¹ |  |  |
| MELD score | 1.01 (0.97 – 1.05) | 0.7 |
| Albumin duration (<24 h vs. 24-60 h) | 1.48 (0.90 – 2.45) | 0.12 |
| Etiology (ref: ALD) |  |  |
| Viral | 1.98 (0.76 – 5.16) | 0.2 |
| MASLD | 2.43 (1.14 – 5.15) | **0.021** |
| metALD | 2.12 (0.91 – 4.94) | 0.081 |
| Autoimmune | 3.55 (1.06 – 11.9) | 0.039 |
| Other | 6.00 (2.78 – 13.0) | **<0.001** |
| Treatment refractory ascites at diagnosis (yes vs. no) | 1.21 (0.68 – 2.14) | 0.5 |
| Sodium | 0.98 (0.93 – 1.03) | 0.4 |
| Albumin | 0.98 (0.93 – 1.02) | 0.3 |
| Platelets | 1.00 (0.99 – 1.00) | 0.3 |

**Supplementary Table 5: Multivariable Cox regression analysis for LT-free survival.**

^1^ Adjusted for MELD, albumin duration, etiology, ascites at diagnosis, sodium, albumin, and platelets Model 1 and 2 include MELD or ACLF together with albumin treatment duration. Model 3 is the fully adjusted model including MELD, albumin duration, etiology of liver disease, ascites at diagnosis, serum sodium, albumin, and platelet count. Hazard ratios (HR) with 95% confidence intervals (CI) are provided.

*ACLF - acute-on-chronic liver failure, MELD – Model of End-Stage Liver Disease, MASLD - metabolic dysfunction–associated liver disease, metALD - MASLD with significant alcohol intake*

**Supplementary Figure 2**

**Supplementary Figure 2: Cumulative incidence of any response to terlipressin therapy (A) and HD- and LT-free survival (B) according to albumin treatment duration (<24 hours vs. 24–60 hours) after propensity score matching.**

The cumulative incidence of complete response to terlipressin treatment did not differ significantly between groups (A: p = 0.4, Gray’s test). No significant difference regarding HD-/ and LT-free survival in patients with any response to terlipressin therapy (B, p = 0.31)

*HD – hemodialysis, LTX - liver transplantation*

**Supplementary Figure 3**

**Supplementary Figure 3: Cumulative incidence of complete response to terlipressin therapy (A) and LT-free survival (B) according to albumin treatment duration (<24 hours vs. 24–60 hours) after propensity score matching.**

The cumulative incidence of complete response to terlipressin treatment did not differ significantly between groups (A: p = 0.8, Gray’s test). Kaplan-Meier curves showing the incidence of LT-free survival (B, p = 0.34) stratified by albumin treatment duration.

*LT - liver transplantation*
